# Morphlight theory inspired by raptors: musculoskeletal modeling and muscle control in *Falco peregrinus* wing flapping

**DOI:** 10.1242/bio.061859

**Published:** 2025-04-10

**Authors:** Di Tang, Kai Chen, Yanbin Dai, Yang Liu, Yibo Zhao, Kunpeng Wang, Siyu Wang, Zhongyong Fan

**Affiliations:** ^1^ College of Mechanical Engineering, Zhejiang University of Technology, Hangzhou 310014, China; ^2^ Department of Life Sciences, Zhejiang Museum of Natural History, Hangzhou 310014, China

**Keywords:** *Falco peregrinus*, Flapping motion, Morphlight theory, Muscle function, Musculoskeletal system, Raptor

## Abstract

*Falco peregrinus* can achieve highly maneuverable flight through their morphing wing structure, which has significant research value. However, there has been limited research on the *F. peregrinus* wing musculoskeletal system. In this study, musculoskeletal modeling, flapping movement, and muscle function of *F. peregrinus* wing were studied through computer modeling and simulation to better understand the biomechanics of *F. peregrinus* wing flapping. Using anatomical data and the musculoskeletal modeling method based on OPENSIM, a three-dimensional model of the *F. peregrinus* wing was developed. Based on the experimental data, the flapping movements were reconstructed, muscle movements during different stages of flapping were simulated, and the function of muscles in the flapping process was analyzed. While this study provides valuable insights into the muscle function of *F. peregrinus* wing during flapping, it also highlights certain limitations, such as the simplification of musculoskeletal structures and joints in the modeling approach and deviations from actual *F. peregrinus* wing movements. This study provides both experimental and analytical methods for raptor wing flapping research, potentially reducing the need for live experiments and offering valuable insights into the mechanisms of raptor flapping.

## Teaser

**Summary:** Musculoskeletal modeling and muscle control in *Falco peregrinus* wing flapping. This study elucidated the function of specific wing muscles in *F. peregrinus*, establishing a novel methodology for raptor muscle research.

## INTRODUCTION

*Falco peregrinus* can achieve maneuverable flight by utilizing its morphing wing structure integrated with the musculoskeletal system. However, limited research has been conducted on computed muscle control of *F. peregrinus* wing musculoskeletal system. Consequently, the computed muscle control of a *F. peregrinus* was quantitatively analyzed to estimate the morphing wing mechanism.

The wing plays a critical role in maneuvering flight in birds; however, the anatomy structure of raptors is rarely reported. Fortunately, avian anatomy has been well investigated in species such as pigeons, which can serve as a good reference. Hieronymus (2016) used rock pigeon wings as samples for observation through standard dissection, micro-computed tomography, and tissue sectioning. They found that abundant smooth muscles play a role in efficiently maintaining folded wing posture, tuning wing shape, and regulating aeroelastic behavior during flight. Additionally, skeletal muscle tissue from the right wing of the rock pigeon was obtained. Meyers (1992, 1996) dissected the shoulder and forearm muscles of the American kestrel, analyzed muscle function in flight by studying the histomorphology, and mapped the location of each muscle in the shoulder and forearm. Tobalske and Biewener (2008) measured muscle length and activity in pigeons using sonomicrometry and electromyography techniques. Dial et al. (1991) used radiography and electromyography to study skeletal offsets and the activity of 11 shoulder muscles in purple-winged starlings. Bribiesca-Contreras and Sellers (2017) performed µCT scanning of sparrowhawk wings and proposed a three-dimensional (3D) model of the sparrowhawk wing musculoskeletal system. However, if the pulley and tendon sheath, composed of connective tissue, are severed during dissection, the pulling direction of the muscles will change, and the observed movement may become inaccurate (Vazquez, 1995). Moreover, conducting live flight experiments on raptors is challenging, and biomechanical data are difficult to measure experimentally, making further simulation studies necessary. Models and simulations can assess how changes in each input (e.g. anatomy, kinematics) independently influence function (Heers et al., 2018).

As many as 36 major pelvic limb muscle groups were incorporated into a 3D biomechanical computational model of the ostrich (Hutchinson et al., 2015). This model was used to investigate the function of the pelvic limb muscles in locomotion, verifying that the ostrich achieves movement through muscle-tendon optimization and static weight support. Subsequently, the location, length, excitation, force, etc. of each muscle are critical parameters for musculature modeling that must be determined in detail. Heers et al. (2018) modeled and simulated the bird musculoskeletal system using OPENSIM, controlled muscle parameters separately, and assessed their influence on movement by calculating muscle control, thereby predicting the functions of different muscles during movement.

In fundamental research on raptors, the mechanisms underlying wing deformation, and their flight capabilities have been observed, and these deformable wings are referred to as morphing wings (Tang et al., 2023, 2024). We integrate the shape-shifting ability and flight capabilities to propose an original scientific theory, Morphlight Theory, in which the *F. peregrinus* wing musculoskeletal system and muscle control are key components. *F. peregrinus* exhibits superior flight performance compared to other birds, maintaining high maneuverability even at elevated speeds. This maneuverability necessitates greater skeletal stability and a more advanced musculoskeletal structure; however, limited research and experiments have been conducted. Therefore, a detailed analysis of the wing musculoskeletal system and the corresponding computed muscle control is necessary to estimate the super-maneuvering mechanisms of raptors.

**Fig. 1.**
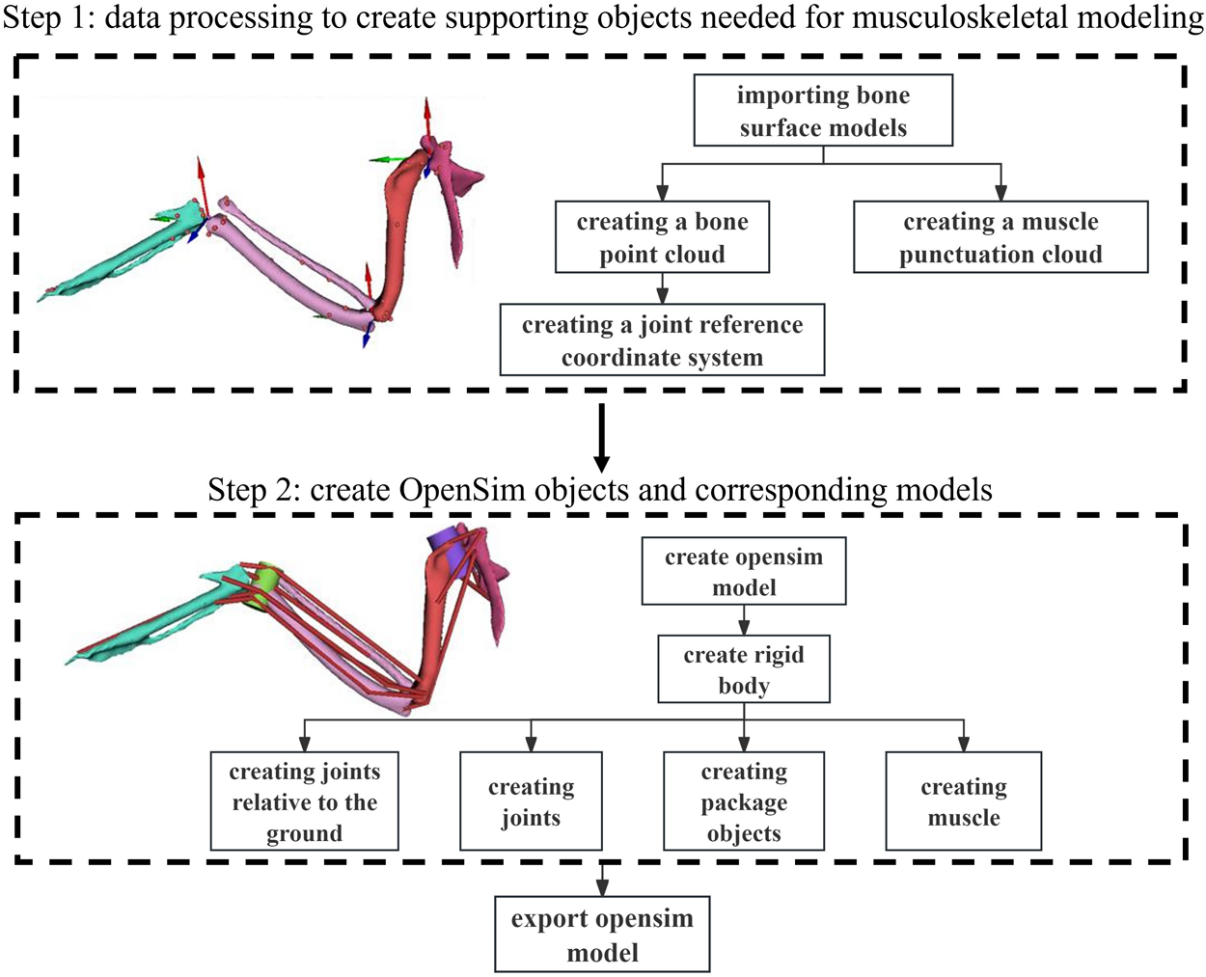
Steps of musculoskeletal modeling for the *F. peregrinus*’ left wing.

## RESULTS

### CT scan and reconstruction of musculoskeletal system

**Fig. 2.**
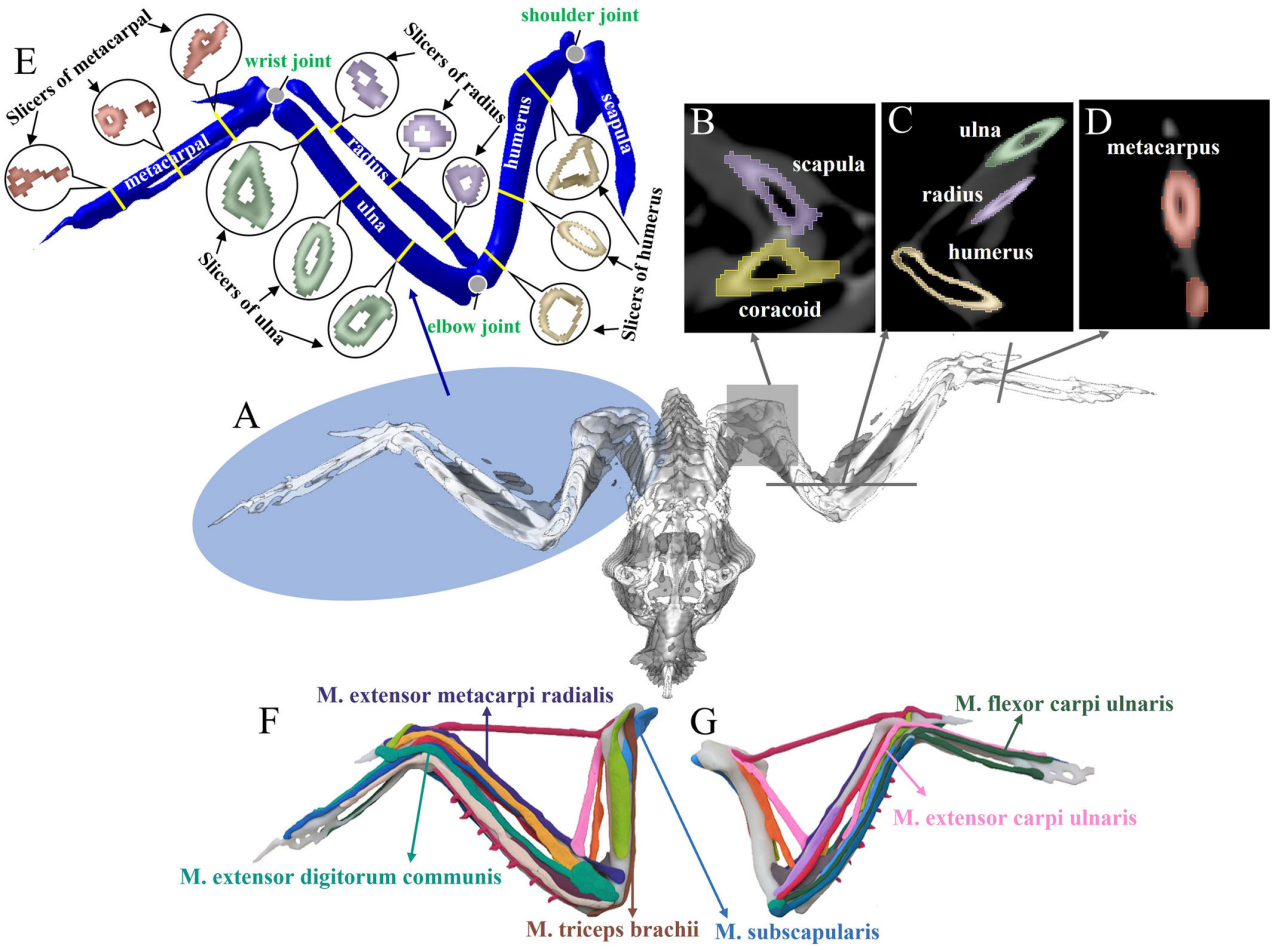
**CT scans of the wing skeleton bones and muscles illustrated.** (A) CT appearance and muscle features. (B) Cross-section of the shoulder joint. (C) Cross-section of the elbow joint. (D) Cross-section of the wrist joint. (E) Skeletal morphology of the metacarpal, radius, ulna and humerus of the *F. peregrinus’* left wing. (F) Manual musculoskeletal model. Each muscle represented with different colored clay was kneaded from the proximal end to the distal end to achieve a more intuitive understanding of the muscle’s morphology. Dorsal view. (G) Ventral view.

The axial, sagittal, and coronal sections of the bone were obtained through CT scanning (Fig. 2A-D). The mass of each bone in the wing skeleton was measured (Table S1). Smoothing algorithms were applied to reconstruct the bone with high precision, and cross-sections of the four main wing bones (humerus, ulna, radius, and metacarpus) were analyzed at the proximal, middle, and distal ends. As a result, a detailed 3D model of the *F. peregrinus* wing skeleton was obtained (Fig. 2E).

The six selected muscles were dissected to gather key information about their origin, insertion and attachment points, and muscle length (Table 1). These data are derived from our experimental measurements. Proximal refers to the side of the bone closest to the body, while distal refers to the side farthest from the body.

**Table 1.** The starting point, insertion point position of muscles and muscle length

Muscle name	Proximal end – distal end	Maximum muscle length (mm)	Minimum muscle length (mm)
M. subscapularis	scapula - proximal humerus	14.6	13.7
M. scapulotriceps	scapula - proximal radius	74.9	73.4
M. extensor digitorum communis	distal humerus - proximal metacarpal	139.0	136.7
M. flexor carpi ulnaris	distal humerus - proximal metacarpal	105.9	103.2
M. extensor metacarpi radialis	distal humerus - proximal metacarpal	97.9	89.9
M. extensor carpi ulnaris	radius - metacarpal	105.3	100.3

Based on the muscle location data from the CT scan and anatomy information, as well as ventral and dorsal views of the muscle tissues, bionic muscles were mounted on the 3D-printed skeletal model using Play-Doh colored clay (Fig. 2F,G). 1.75 mm white T-PLA resin was used as the 3D printing material. Blue clay substitutes for M. subscapularis, and brown clay substitutes for M. scapulotriceps, both originating from the scapula. M. subscapularis terminates at the proximal end of the humerus, and M. scapulotriceps terminates at the proximal end of the radius. Light green clay represents M. extensor digitorum communis, dark green clay represents M. flexor carpi ulnaris, and purple clay represents M. extensor metacarpi radialis, all originating from the distal end of the humerus to the proximal end of the metacarpal. M. extensor carpi ulnaris is represented by pink clay, extending from the radius to the metacarpal.

### Measurement and reconstruction of flapping motions

The residuals for each case were computed using the optimization algorithm (Table S2). The values of these residuals are derived from calculations. Where S represents a spherical hinge, U represents a universal joint, and R represents a revolute joint. The lower the residuals, the higher the computational accuracy achieved. The residuals of the spherical-spherical joint reached their minimum value due to its high degrees of freedom. As a balance between bionic accuracy and complexity, the revolute-universal joint was selected for the flapping study, and the kinematic vice was modeled as a two-degree-of-freedom joint with a residual of 0.5932 mm. The optimal computational results were selected as follows: the rotational center of the shoulder joint was optimized to −11.2, 0.1, 0.1, and the rotational center of the wrist joint was optimized to −17.3, 81.4, 39.2.

**Fig. 3.**
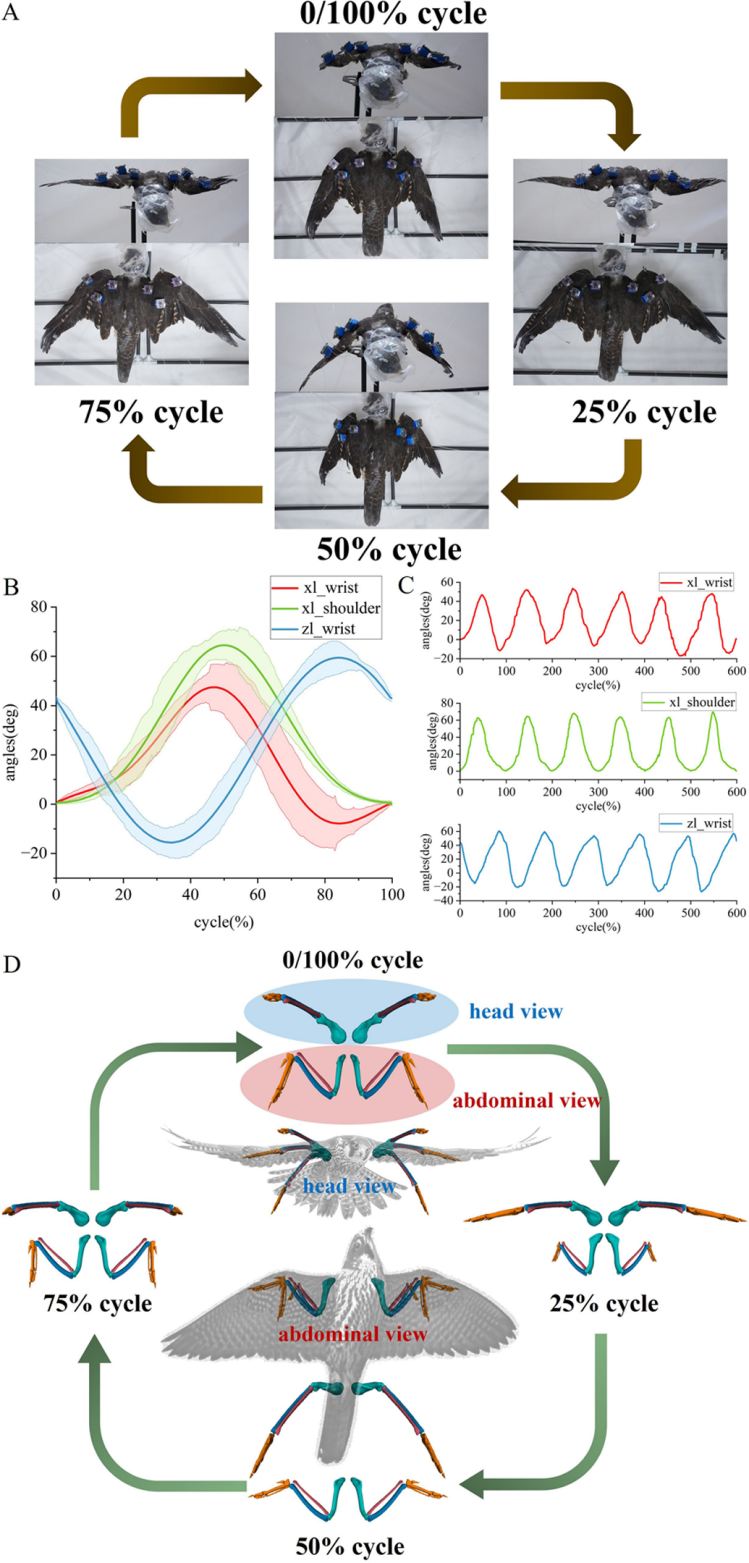
**(A) Experimental arrangement of *F. peregrinus*.** The specimen was suspended in the suspension system with the sensor mounted. Wing movements of *F. peregrinus* at different flapping stages were recorded. (B) Measured wing flapping motions in a period after fitting. The three smooth curves represent the flapping wing motion angles fitted by Fourier series, and the shaded parts around the curves are the deviations. (C) Wing flapping rotation angles measured by angle sensors. (D) Dynamic anatomy of *F. peregrinus* during a flapping wing cycle from different perspectives, showing the skeletal morphology at 0/100%, 25%, 50%, and 75% of the cycle.

The joint rotation angles of *F. peregrinus* flapping wing motion were accurately measured and digitized (Fig. 3B,C). The joint rotation angles of *F. peregrinus* flapping wing motion are derived from experimental measurements. The flapping motion of the shoulder joint is denoted as xl_shoulder, while the wrist joint flapping motion is denoted as xl_wrist. Additionally, the extension and flexion movements of the wrist joint are denoted as zl_shoulder. In this study, the motion of the wing joints is represented using a Fourier series, and the flapping angle and its associated deviation band within a complete flapping cycle are obtained (Fig. 3B). Previous studies, such as Liu et al. (2006), have demonstrated the effectiveness of using Fourier series to approximate the motion of wing joints. This method is particularly useful for modeling periodic motions where precise, continuous data from live specimens may not always be available. Bird wing joints may not exhibit perfectly smooth, repetitive motion in all situations. However, the Fourier series provides a reasonable approximation given the available data. The flexibility of the Fourier series allows it to model the periodic nature of wing flapping while accounting for the variability in the motion pattern. This approach strikes a balance between model simplicity and the need for accuracy, making it a useful tool for simulating wing joint dynamics in the absence of more complex motion data.

During the 0-50% of the cycle, the shoulder joint exhibits a downward flapping motion. Conversely, the shoulder joint moves upward during the terminal 50-100% of the cycle. This flapping motion has an angular amplitude of 64°. On the other hand, the wrist joint flaps downward during 0-47% and 84-100% of the cycle, and flaps upward during 47-84% of the cycle. Meanwhile, a 75° rotation was also found for zl_wrist, indicating that the outer wing was not only flapped but also folded simultaneously during a flap cycle. The outer wing folds during the 34-84% of the cycle. This biomechanical observation can be further used to analyze the physiological characteristics of *F. peregrinus*.

The angle of flapping wing motion was converted into OPENSIM motion files, which were then used to drive the musculoskeletal model to flap according to the set angle data. This not only replicated the wing gestures of the *F. peregrinus* but also captured dynamic flapping motions of the wing (Fig. 3D). Cycle at 0%, 25%, 50%, and 75% were selected as critical frames to dynamically analyze the movement process of *F. peregrinus* wings during a flapping cycle. Both head and abdominal views were presented to illustrate the musculoskeletal gestures.

### Model validation

**Fig. 4.**
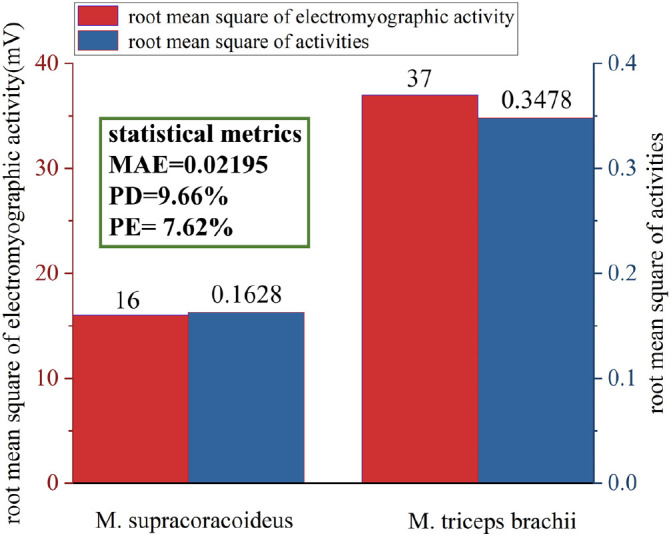
Comparison of experimental (Meyers, 1993) and simulated muscle activity.

To validate the established musculoskeletal model, we compared the root mean square (RMS) of *F. peregrinus*’ muscle activities, calculated via the computed muscle control method, against experimental values from the American kestrel (Fig. 4). Meyers (1993) studied the root mean square of electromyographic activity in the shoulder muscles of the American kestrel (*Falco sparverius*), specifically M. supracoracoideus and M. triceps brachii, during wind tunnel flights. *F. peregrinus* and *F. sparverius*, both members of the genus *Falco*, share a close evolutionary relationship. Anatomical investigations have found significant similarities in the muscle physiology of both species. Therefore, the primary functions of M. supracoracoideus and M. triceps brachii during flapping motion can be considered analogous between the two species, providing a robust basis for comparing their electromyographic signals. For quantitative analysis of muscle functions, the root mean square of the two muscle activities computed in this study was compared with the root mean square of electromyographic activity of the two muscles (Meyers, 1993). Muscle activity data were obtained from OPENSIM 4.3 simulations, whereas electromyographic activity data were sourced from Meyers (1993). To quantitatively assess agreement, we normalized both the simulated root mean square values of *F. peregrinus*’ muscle activities (16 and 37) and the electromyographic root mean square values (0.1628 and 0.3478), subsequently calculating key statistical metrics. Mean absolute error (MAE), percentage difference (PD), and proportional error (PE) metrics were employed to evaluate model accuracy. The results were: MAE=0.02195, PD=9.66%, and PE=7.62%. These results indicate that both the computed myoelectric signals and the measured electromyographic activity accurately represent muscle functions. Additionally, the OPENSIM musculoskeletal model of the *F. peregrinus* wing was established, and its accuracy was verified.

### Shoulder muscle analysis

**Fig. 5.**
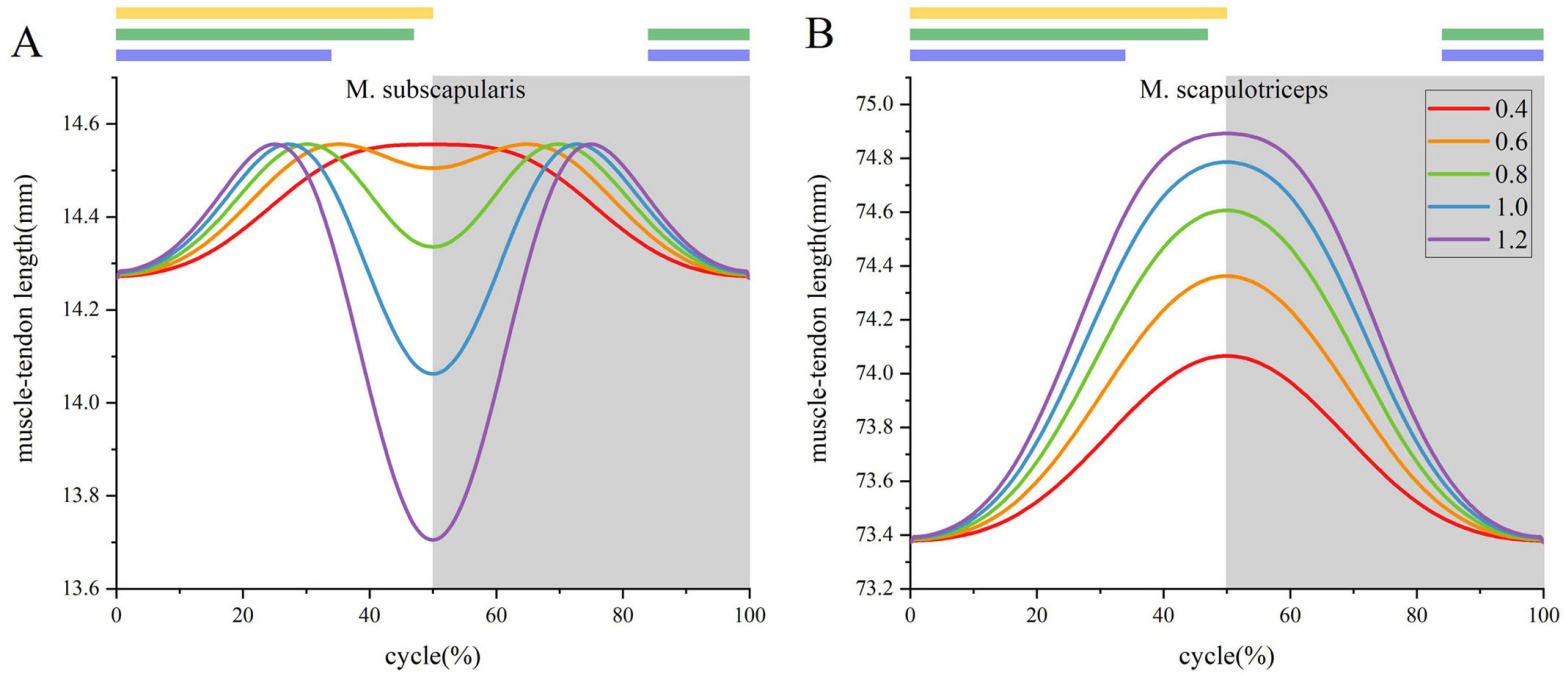
**The muscle-tendon length changes over time during different flapping movements in a cycle.** The gray background indicates the time period during which the muscle-tendon length is contracted, that is, the time period during which the muscle is activated (generating force). The yellow section indicates the downstroke of the humerus, the green section represents the downstroke of the metacarpi, and the purple section represents the extending motion. (A) M. subscapularis. (B) M. scapulotriceps.

To elucidate the intricacies of muscular function in flapping wing dynamics, a comprehensive analysis of muscle-tendon length variations was conducted across a range of flapping amplitudes. The experimental design ensured a constant temporal threshold to achieve peak amplitude at each angular increment during the wing flapping cycle. The flapping amplitudes 0.4, 0.6, 0.8, 1.0, and 1.2 were selected to investigate muscle functions. Notably, the 1.2-fold amplification approximated the upper limit of the angular range achievable by *F. peregrinus* during flapping. Consequently, this amplitude was selected as the upper boundary for our investigation. The resultant data, which encapsulate the temporal progression of two distinct muscles, were presented graphically (Fig. 5). This figure illustrates the intricate relationship between muscle-tendon length and wing flapping kinematics across the varied amplitudes. The muscle-tendon length values were derived from OPENSIM 4.3 simulation results.

**Fig. 6.**
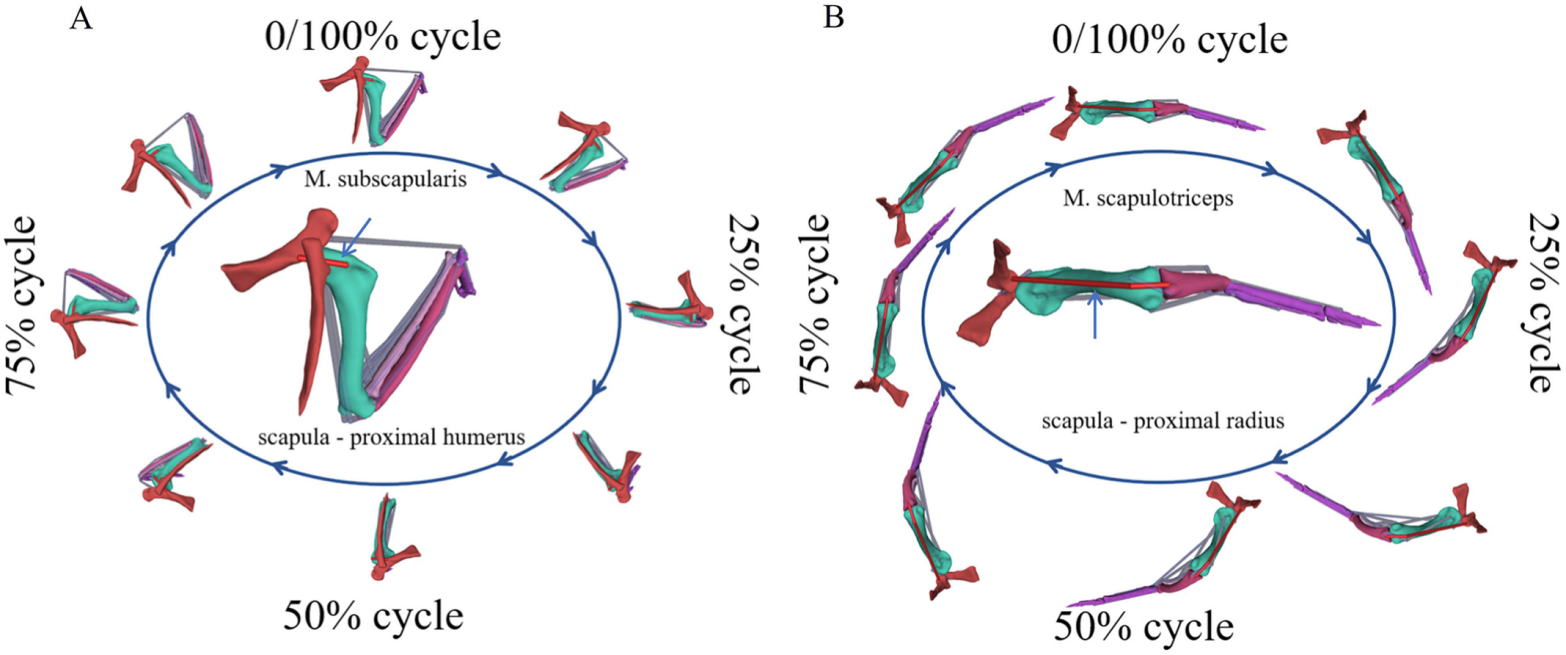
**Static and dynamic anatomy of each muscle during a flapping wing cycle.** The excited muscle is highlighted in red. The ellipse is surrounded by eight frames, showing the muscles morphological changes during a flapping cycle. (A) M. subscapularis. (B) M. scapulotriceps.

To enable clearer observation of muscular alterations at different temporal intervals, the aforementioned muscles were analyzed through both static and dynamic dissection during the entire wing flapping cycle (Fig. 6). This approach allows for a thorough assessment of muscle behavior in distinct phases of wing motion, thereby improving our understanding of their functional dynamics.

### Wrist muscle analysis

**Fig. 7.**
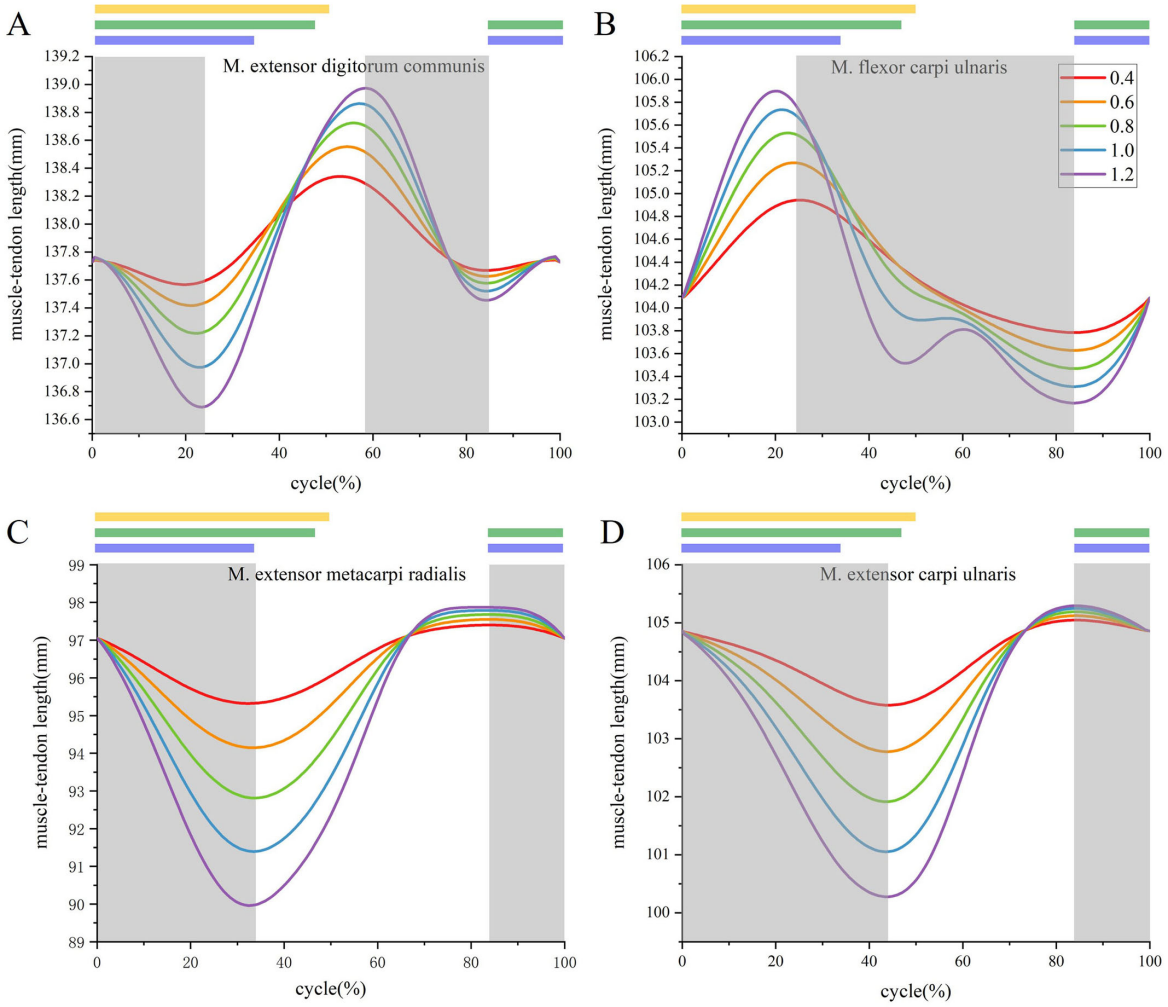
**The muscle-tendon length changes over time during different flapping movements in a cycle.** (A) M. extensor digitorum communis. (B) M. flexor carpi ulnaris. (C) M. extensor metacarpi radialis. (D) M. extensor carpi ulnaris.

**Fig. 8.**
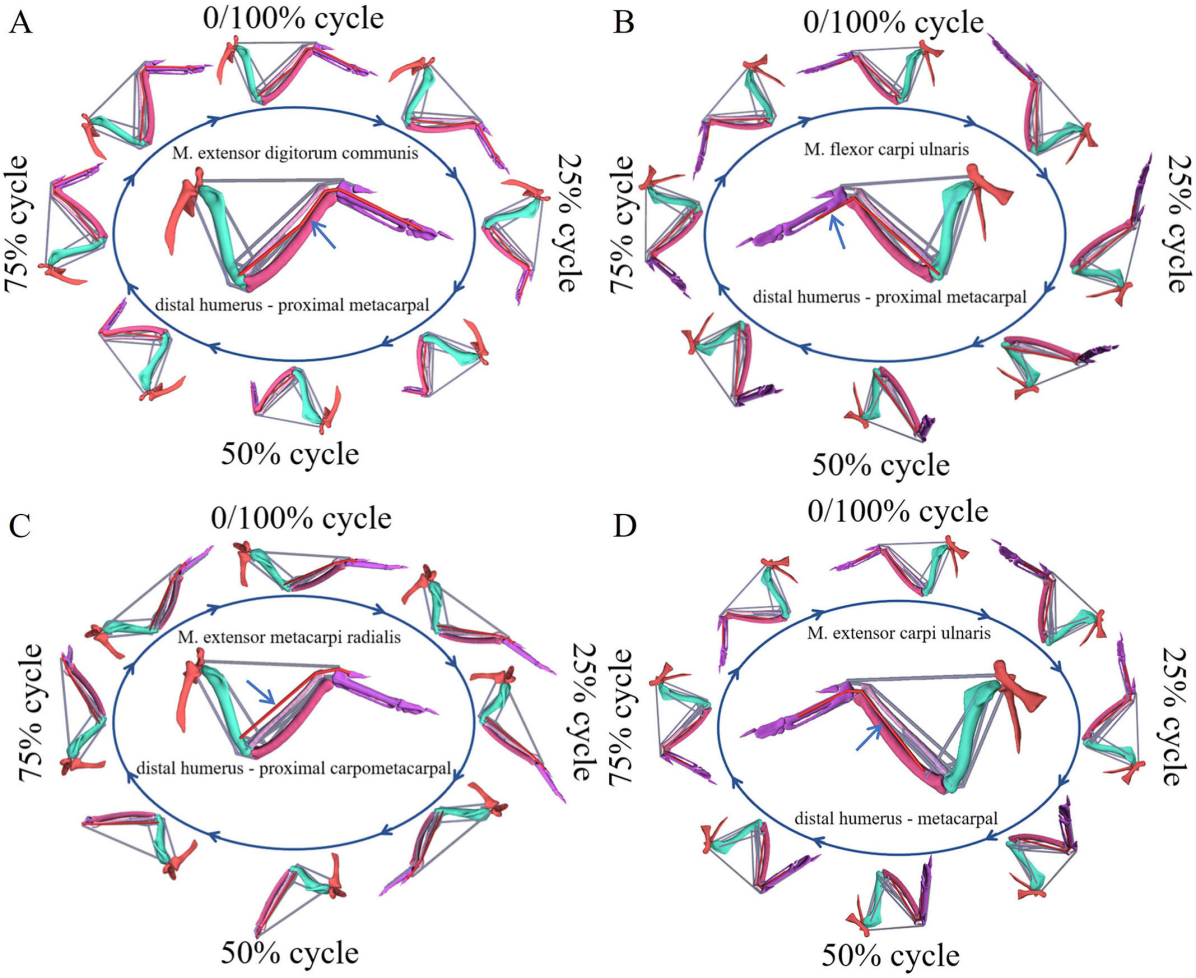
**Static and dynamic anatomy of each muscle during a flapping wing cycle.** (A) M. extensor digitorum communis. (B) M. flexor carpi ulnaris. (C) M. extensor metacarpi radialis. (D) M. extensor carpi ulnaris.

The wrist joint plays a crucial role in wingbeat, however, limited observations of muscle functions around the wrist joint were found, as they were too small to measure directly in experimental. As an alternative, the computed muscle control results were used to evaluate their performance. Similarly, muscle-tendon length (Fig. 7), static anatomy, and dynamic anatomy (Fig. 8) were further analyzed for the shoulder muscle. The muscle-tendon length values were derived from OPENSIM 4.3 simulation results.

A more intuitive visualization of the muscles and their associated morphological changes during flapping motion has been developed.

### Sensitivity analyses

To assess the robustness and reliability of our musculoskeletal model, we conducted a sensitivity analysis focusing on key biomechanical parameters, notably the optimal fiber length and tendon slack length. We varied these parameters within physiologically realistic ranges to observe their impact on model predictions. Each parameter was altered by ±20%, ±40%, and ±60% from its baseline value, and simulations were rerun to evaluate changes in muscle-tendon length. The muscle-tendon length remained consistent across parameter variations of ±20%, ±40%, and ±60%. When the optimal fiber length was reduced by 60%, the change in muscle-tendon length was less than 0.1‰, whereas a 60% reduction in tendon slack length resulted in a change of less than 0.1‰. This is because muscle-tendon length is influenced by muscle path, skeletal geometry, and joint motion (Delp et al., 1990; Dvinskikh et al., 2010). These results suggest that our model is stable within reasonable ranges of parameter uncertainty.

In addition to the sensitivity analysis, we further explored how muscle-tendon length varied within a single flapping cycle across different motion amplitudes of the same movement type. This analysis aimed to assess the sensitivity of our model to changes in motion amplitude, a critical factor in dynamic biomechanical simulations. The results showed that muscle-tendon length exhibited consistently similar trends across different motion amplitudes, and the muscle contraction period was highly coincident with each other (Table 2), confirming consistency in our model's predictions. Due to the unique geometry of the skeletal structure and the specific muscle paths of M. scapulotriceps and M. extensor carpi ulnaris, these two muscles exhibited less change in their activation periods when the motion amplitudes was altered. This consistency in muscle-tendon length response under varying conditions. Above all, the musculoskeletal model's reliability and parameter sensitivities were irrefutable verified.

**Table 2.** Sensitivity analysis across different motion amplitudes

Muscle name	Motion amplitudes	Muscle contraction period (%)	Average error
M. subscapularis	0.4	50-100	/
	0.6	35-50, 65-100	8%
	0.8	30-50, 70-100	3%
	1	27-50, 73-100	/
	1.2	25-50, 75-100	2%
M. scapulotriceps	0.4	50-100	<0.01%
	0.6	50-100	<0.01%
	0.8	50-100	<0.01%
	1	50-100	/
	1.2	50-100	<0.01%
M. extensor digitorum communis	0.4	0-19.5, 53.5-84	3.5%
	0.6	0-21, 55-84	2%
	0.8	0-22, 56-84	1%
	1	0-23, 57-84	/
	1.2	0-23.5, 58.5-84	1.5%
M. flexor carpi ulnaris	0.4	25.5-83.5	4%
	0.6	24-83.5	1.5%
	0.8	22.5-83.5	1%
	1	21.5-83.5	/
	1.2	20-83.5	1.5%
M. extensor metacarpi radialis	0.4	0-32.5, 84-100	0.75%
	0.6	0-33.5, 84-100	0.25%
	0.8	0-33.5, 84-100	0.25%
	1	0-33.5, 83.5-100	/
	1.2	0-32.5, 82-100	1.25%
M. extensor carpi ulnaris	0.4	0-44, 85-100	0.5%
	0.6	0-43.5, 84.5-100	<0.01%
	0.8	0-43.5, 84.5-100	<0.01%
	1	0-43.5, 84.5-100	/
	1.2	0-43.5, 84.5-100	<0.01%

## DISCUSSION

### Muscle function

The M. subscapularis is positioned cranially to the scapulohumeralis caudalis and spans from the scapula to the humerus, caudal to the shoulder joint (Fig. 6). This anatomical configuration is crucial for its function. Intriguingly, the muscle-tendon length variation of the M. subscapularis during wing flapping exhibited distinct patterns, possibly due to the M. subscapularis starting to bypass the wrapper object. Activation of the M. subscapularis occurs during the shoulder flapping phase, suggesting its significant role in shoulder flapping during wing motion. This function aligns with the description of the M. subscapularis in American kestrel by Meyers (1992), who utilized the retractor of the humerus to elucidate its role.

The M. scapulotriceps, a muscle extending from the scapula to the ulna, caudal to the shoulder joint (Fig. 6), exhibited activation during the shoulder overthrow phase of movement. This temporal correlation with the shoulder's kinematic profile suggests a mechanistic role for the M. scapulotriceps in shoulder overthrow, consistent with Biewener's (2011) analysis of M. scapulotriceps of pigeons.

The M. extensor digitorum communis extended from the distal humerus to the alula and major digit. Its apex attaches to a process on the lateral epicondyle of the distal humerus, between the origins of the M. extensor carpi radialis and the M. extensor carpi ulnaris. The tendon emerged from the sulcus at the distal end of the carpometacarpus, passed through a tendon loop, and made a 90-degree turn ventrally between the carpometacarpus and the proximal phalanx of the major digit. It then passed deep to the sesamoid in the tendon of the M. extensor longus digiti majoris. The M. extensor digitorum communis inserted onto the proximal cranio-ventral corner of the proximal phalanx of the major digit (Meyers, 1992) (Fig. 8). This muscle is activated during the wrist's downward and extension phases, suggesting that the M. extensor digitorum communis plays a significantly role in the wrist's downward and extension during the flapping cycle. This observation is consistent with the findings of Bribiesca-Contreras et al. (2021), who identified the role of this muscle in wrist flexion in aquatic birds.

The M. flexor carpi ulnaris originates from the medial epicondyle of the humerus and extends distally toward the wrist (Fig. 8). During the contraction phase of wrist joint movement, the M. flexor carpi ulnaris shows prominent activation. This suggests that the M. flexor carpi ulnaris plays a key role in mediating wrist flexion. The temporal alignment of the M. flexor carpi ulnaris activation with the wrist joint's contractile phase indicates a direct mechanistic contribution to the flexion movement.

The musculotendinous unit of the M. extensor metacarpi radialis, a prominent antebrachial muscle in the genus *F. peregrinus*, is located along the cranial aspect of the forearm (Fig. 8). Notably, a decrease in the length of this musculotendinous unit occurs during the initial 0-34% and terminal 84-100% phases of the wing flapping cycle, indicative its active involvement during these phases. This suggests a key role for the M. extensor metacarpi radialis in wrist joint extension during wing flapping. The analysis of the function of M. extensor metacarpi radialis and M. flexor carpi ulnaris in wing flapping is consistent with the findings of Vazquez (1994) in pigeons.

The M. extensor carpi ulnaris, a muscle with parallel fibers, is located on the caudal border of the dorsal antebrachium. It lies deep to the M. extensor digitorum communis and spans from the distal radius to the proximal metacarpus (Fig. 8). Given its anatomical position and activation pattern, it is hypothesized that the M. extensor carpi ulnaris plays a crucial role in wrist flapping and extension during the wing flapping.

### Model limitations and future work

*F. peregrinus* is classified as a second-class protected animal in China, where any sale, purchase, or use of this wild species is strictly prohibited. Future research, under proper authorization, could involve a larger sample size to enhance the robustness and generalizability of the findings. Additionally, we believe the data obtained from this specimen provide valuable insights into the musculoskeletal mechanics of *F. peregrinus*.

Due to the absence of direct EMG data for *F. peregrinus*, we compared our computed muscle activity with experimental data from the *F. sparverius*. We acknowledge differences between the two species in body size, wing morphology, flight dynamics, and muscle properties, all of which may influence muscle activation patterns. To account for these differences, we applied normalization techniques in our model. However, we acknowledge the inherent limitations of this cross-species comparison. Future research will aim to obtain species-specific EMG data for *F. peregrinus* to further validate and refine the model.

Given the complexity of the anatomical intricacies of raptors and the limitations of the modeling approach, it is important to acknowledge the simplifications made in the model. Specifically, during the modeling process, the scapula and coracoid were treated as rigid bodies, and the wings were simplified into a two-bar model, which has been shown to be reasonable in previous studies (Tang et al., 2023; Liu et al., 2006), although deviations remain when compared to real birds. Additionally, the joint mechanics used in this study are idealized and based on revolute joint models. In real birds, joint rotation may involve more complex interactions than those accounted for by a single-axis revolute joint. Especially raptors like the *F. peregrinus*, have highly specialized joint, muscle antagonism and bone structures that may not be fully captured by the foundational human joint models in OPENSIM. This study does not consider the nonlinearities in biological joints, such as cartilage deformation and ligament elasticity, which may influence muscle activity. These simplifications inevitably affect the accuracy of the simulations. Fortunately, Heers et al. (2018) did not incorporate the nonlinearities of joint mechanics in their study either. However, their simulation results were highly consistent with the muscle activity of real birds, leading us to hypothesize that these factors have minimal impact on muscle activity. Furthermore, this study reconstructed the flapping motion with the guidance of ornithologists to ensure accuracy. However, we did not perform control tests on alternative subjects, such as non-raptor species. One limitation of the flapping experiment is that the motion of the sample differs from its natural state; only the geometric pose data has reference value, and dynamic information such as acceleration and frequency cannot be reproduced through a static specimen. More valuable dynamic information needs to be collected in future studies involving live samples (e.g. pigeons, parrots) for a more comprehensive understanding. This will allow for the collection of reliable dynamic data such as acceleration and frequency. Therefore, the model in this study is limited to the flapping motion of *F. peregrinus* wing under idealized joint mechanics and does not fully represent the dynamic flight patterns exhibited by real birds, particularly under varying environmental conditions. Future work should incorporate more complex joint mechanics and anatomical differences to improve the accuracy of the simulations and better simulate the biomechanics of raptor flight.

### Conclusions

*F. peregrinus* possesses exceptional maneuverability due to its unique musculoskeletal system. However, limited research has been conducted on its muscle function, leaving its flight capabilities largely unexplained. Therefore, we developed a numerical musculoskeletal model to investigate the underlying mechanisms of its flight. In this study, we present the following findings:

1. A wing musculoskeletal system was established, and a computed muscle control method was introduced to further analyze wing motion and muscle function during a flapping cycle.
2. The flapping angle of the *F. peregrinus* wing was measured to replicate the wing flapping motion, which is valuable for studies on bird flapping.
3. Muscle activity and muscle-tendon length were quantitatively assessed to analyze muscle function during a flapping cycle. The M. subscapularis and the M. scapulotriceps contribute to supra-shoulder flapping during wing flapping. The M. supracoracoideus plays a crucial role in the wing beating. The M. extensor digitorum communis contributes to wrist downward and extension during flapping, while the M. extensor metacarpi radialis facilitates wrist extension. M. extensor carpi ulnaris and M. flexor carpi ulnaris, functioning as antagonist muscles, play a significant role in wrist flexion.

We believe our observations provide both experimental and analytical methods for raptor flapping research and offer novel insights into the musculoskeletal system of the *F. peregrinus*. By understanding specific muscle functions during flapping, we can enhance our understanding of avian locomotion. This study has implications for the biomechanics of raptor wing flapping and muscle function.

## MATERIALS AND METHODS

### CT scan and muscle anatomy

The CT scanning experiment was conducted and discussed in our previous research (Tang et al., 2023). We performed CT scans of *F. peregrinus* using the GE Optima 540 16-slice CT scanner. Subsequently, the bone mass of each wing skeleton was measured, and the scanned DICOM data were processed using the 3D SLICER. Each bone, muscle, and feather were identified using density thresholds, then segmented, isolated, and reconstructed. The relatively small-sized bones of the *F. peregrinus*, with a wingspan of 0.8 m may contribute to this deviation. Subsequently, smoothing algorithms were applied to reconstruct the bones with high precision. Cross-sections of the four major wing bones (humerus, ulna, radius, and metacarpal) were examined at the proximal, middle, and distal ends. As a result, a refined 3D model of the *F. peregrinus* wing skeleton was obtained.

To study the wing flapping of a *F. peregrinus*, the wing skeleton model was simplified to a two-bar model with two joints. Therefore, the rotations of both the shoulder and the wrist joint were considered. Previous research (Liu et al., 2006) has shown that using these critical degrees of freedom to reconstruct a bird's flapping kinematics is reasonable. Thus, in the subsequent biomechanical study, only the muscles influencing the shoulder and wrist joints were considered. The muscles of the *F*. *peregrinus* wing were screened, and a total of six muscles around the shoulder and wrist joints were selected. The wing muscles of the *F*. *peregrinus* were dissected to investigate the functions of these muscles.

### Computed muscle control method and OPENSIM modeling

A muscle generates an active force up to a maximum value, depending on its fiber length and activity in the contractile element. In contrast, the generated passive force depends on the fiber length of the parallel element. Finally, the active force and passive force constitute the total muscle force. Therefore, the optimal fiber length, pennation angle, and tendon slack length should be well defined, as they are critical for computed muscle control simulation of *F. peregrinus*.

Optimal fiber length refers to the specific length at which a muscle fiber or a muscle-tendon unit operates with maximum mechanical efficiency (Heers et al., 2018). The pennation angle is the angle formed between the muscle fibers and the line of action of the muscle force. Muscle fibers attach to a tendon at a pennation angle, which scales the force they transmit to the tendon. To measure the optimal fiber length and pennation angle of the *F. peregrinus* wing, the muscles were photographed, and the pennation angle and length of all the muscle fibers were measured using ImageJ. For parallel fibers, the pennation angle was 0 degrees, and fiber length was measured at the midpoint of the muscle. For the pinnated fibers, measurements were taken at both ends of the tendon. The pennation angle was qualitatively consistent with the results of *F. peregrinus* forelimb muscles (Meyers, 1992, 1996).

Tendon slack length (*L*_*ts*_) is defined as the length at which the tendons associated with a muscle begin to resist stretch and generate force. This parameter is crucial because it delineates the extent to which the force exerted by a muscle-tendon unit is actively produced by muscle contraction and passively by the tendon's series elastic component during stretch. The algorithm provided by Manal and Buchanan (2004) was used to calculate the tendon slack length for each muscle. The minimum and maximum lengths of both muscle and tendon (*L*_*mt*_=muscle length *L*_*m*_+tendon length *L*_*t*_) were measured during joint motions, and the average pennation angle α of the muscle fibers along with the normalized fiber lengths were calculated.

The computed muscle control method is an effective approach to obtain biomechanical data, such as muscle activity and muscle force. In this method, muscle activity is modeled by relating the time rate of change of muscle activity (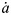) to the muscle activity (*a*) and excitation (*u*):
(1)

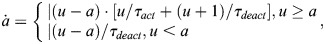
where *τ*_*act*_ and *τ*_*deact*_ are the time constants for activity and deactivation, respectively (Raasch et al., 1997). Musculotendon contraction dynamics are described by a lumped-parameter model that accounts for the muscle's force-length-velocity properties and the tendon's elastic properties (Zajac, 1989). The primary issue is predicting the accelerations of the generalized coordinates in response to applied forces, which can be calculated using the equations of motion:
(2)

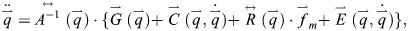
where 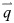, 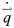, and 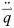 are the generalized coordinates, velocities, and accelerations of the model, respectively, 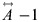 is the inverse of the system mass matrix, 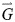 is a vector of generalized forces due to gravity, 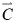 represents the generalized forces arising from Coriolis and centripetal forces, 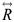 is a matrix of muscle moment arms, 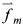 is a vector of muscle forces, and 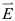 is a vector of generalized forces that characterizes the model's interaction with the environment. In particular, the time rate of change of muscle length 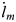 is a nonlinear function of muscle length *l*_*m*_, muscle-tendon actuator length *l*_*mt*_, and muscle activity *a*.

The properties of the musculoskeletal system and its interactions with the environment are described by a set of ordinary differential equations, a forward dynamic simulation is performed using there equations. Feedforward and feedback control were introduced to guide the kinematic trajectory of dynamic model toward a set of measured experimental kinematics The computed muscle control algorithm consists of four calculation steps.

In the first stage, a set of desired accelerations of the generalized coordinates 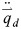 is calculated based on experimental kinematics and the current kinematic state of the model:
(3)

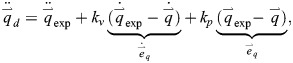
where 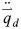 and 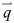 are the generalized velocities and displacements of the model, 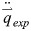, 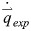 and 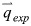 represent the experimental accelerations, velocities, and displacements corresponding to the generalized coordinates of the model, *k*_*v*_ and *k*_*p*_ are the feedback gains for the velocity deviation 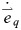 and the displacement deviation 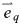, respectively.

In the second stage, an optimization problem is solved to compute a set of muscle activities 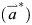 that correspond to the required muscle forces. Subsequently, the desired accelerations 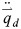 were computed in the first stage, are generated. To estimate the previous acceleration, steady-state muscle forces 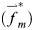 are computed from the muscle activity by considering the force-length-velocity properties of the muscles. These steady-state forces are then applied to the model, and the equations of motion for acceleration are solved accordingly:
(4)

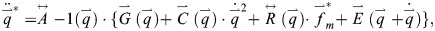
where the asterisks on 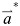, 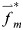, and 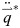 are used to differentiate these steady-state quantities from the corresponding quantities during the forward simulations.

In the third stage, a linear proportional feedback controller is used to compute the excitation that drives muscle activity 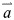 to track 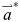:
(5)

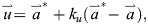
where *k*_*u*_ is the feedback gain, 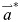 is the activity vector calculated in the second stage, and 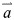 is the current activity vector.

In the fourth stage, muscle excitation is introduced into the forward dynamic model, and the numerical integration approach is used to advance the state to the next time step. By looping through stage 1 to 4, muscle dynamic properties can be computed.

Path information, weight, and length were recorded to construct numerical muscles according to the CT experiment of *F. peregrinus* wing muscle from our previous research (Tang et al., 2023), as well as the anatomical data from Meyers (1992, 1996). Initially, data processing was performed to create the supporting objects required for musculoskeletal modeling, followed by the creation of OPENSIM objects and corresponding models (Fig. 1). Specifically, this process can be divided into the following nine parts:

1. Import bone surface model. CT scan data were used to generate 3D surface models of each bone for defining the model.
2. Create skeleton point cloud. Skeleton points at the joints define the reference coordinate system for the shoulder and wrist joints. In the global coordinate system, the shoulder joint is located at −11.2, 0.1, 0.1 and the wrist joint at −17.3, 81.4, 39.2.
3. Create joint reference coordinate system. Two reference coordinate systems are created for each joint, one for the parent and one for the child. These are aligned with the global coordinate system and do not undergo additional rotation.
4. Create muscle points. Muscle paths are defined by key points (origin, insertion, and intermediate attachment), forming the geometric shape of the muscle.
5. Create rigid bodies. The scapula, coracoid, humerus, ulna, radius, and metacarpus surfaces are modeled as rigid bodies. Input the corresponding bone mass, and the inertial characteristics are automatically calculated by the software and stored in the VME.
6. Define ground-relative joint. The shoulder joint is defined as fixed relative to the ground.
7. Create joints. The shoulder, elbow, and wrist joints are defined with appropriate parent-child body relationships and specified joint types (hinge, fixed, and universal).
8. Create wrap object. Wrap objects (e.g. spheres, ellipsoids, cylinders) are placed on the bone surfaces to prevent muscle crossing during movement.
9. Create muscles. Muscles are created based on their geometric paths, defined by the origin, insertion, and attachment points. OPENSIM muscle objects are generated from the data.

### Flapping motion reconstruction method

A motion optimization algorithm based on the skeletal point cloud was established (Hutchinson et al., 2015) and was subsequently used to analyze the degrees of freedom of the flapping wing skeleton. Subsequently, various bionic joints were used to represent the natural wing skeleton joints, replicating wing flapping motions. In the model, the coracoid and scapula were included to represent the body skeleton (fixed, global frame), and rotations of the humerus, ulna, radius, and metacarpal bones were calculated relative to the global frame. The wing postures, including extension, upward flap, and downward flap, were categorized as j=1∼3 in the quantitative analysis. The humerus, ulna, and metacarpal were denoted as i=1, 2 and 3, respectively. The radius was not considered in the current research. The shoulder joint, elbow joint, and wrist joint were denoted as k=1, 2, and 3. The proximal frame Ap(i) and distal local frame Ad(i) were also attached to each bone to describe its motions relative to adjacent bones.

Similarly, the motions (both axis and angular) of the ulna relative to the humerus and the rotations of the carpometacarpus relative to the ulna, were measured using a step-by-step point cloud fitting approach. Additionally, the motions of the humerus relative to the scapula were also measured using the step-by-step point cloud fitting approach to represent shoulder joint rotations. The average distance between different point clouds was calculated, and the optimization algorithm, utilizing the global optimization method Multi-Start in MATLAB software, was employed to measure the relative rotations during flapping.

In this paper, a point cloud-based skeletal motion optimization algorithm was further developed and applied to the scanned point cloud data. This study focused solely on the rotational motion of the skeleton when analyzing the wing flap motion, and all joint motions adhered to the point constraints. The wing postures were ranked as *j*, and the bones were labeled as *i*. The rotation *R*_*i*_ and translation *T*_*i*_ were used to represent the rotations and transformations of bone *i* in the global coordinate system. The current position *L*_*i*,*curr*_ of the bone *i* can be calculated using the following equation:
(6)

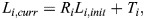
where subscript *init* and *curr* represent the initial and current locations. Therefore, the average distance (i.e. the residual) between the transformed position and the current position can be calculated as:
(7)

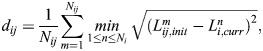
where, subscript *ij* denotes bone *i* in posture *j*.

Typically, each bone is treated as a rigid body to form a multi-body system. In the system, neighboring parts are connected by various types of sub-joints, modeled using constraint equations that include both translational and rotational constraints. Assume that a joint connects the *i*th body and the *j*th body at point m and point n, respectively. Location constraints are established at points m and n, while the z-axis of one joint remains parallel to that of the other. The translational constraint equations are as follows:
(8)

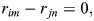
where *r*_*im*_, *r*_*jn*_ represent the locations of point *m* and *n*, respectively, in the global coordinate system. The vector constraint equations are formulated to satisfy the relative angular constraint between the two neighboring bones, as shown in Eqn 9:
(9)

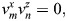
where 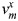 represents the *x*-vector at point *m*, and 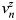 represents the *z*-vector at point *n*. This equation indicates that the joint rotation always satisfies the condition that the z-axis and x-axis are orthogonal to each other.

Combining translational constraint and vector constraint, the equations of the fixed joint are shown in Eqn 10:
(10)

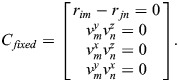
Similarly, the constraint equations for a revolute joint are given by:
(11)

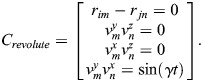
The constraint equations for a universal joint are given by:
(12)

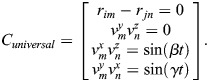
The constraint equations for a spiral joint are given by:
(13)

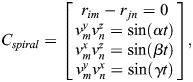
wherein α, β and γ represent the angles of rotation of the joint about the x-axis, y-axis, and z-axis, respectively.

Finally, combine the minimized function and the constraint function to establish the global optimization algorithm as shown in Eqn 14:
(14)

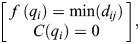
where *q*_*i*_=[*Center*_*i*_, *Direction*_*i*_, *Rotation*_*i*_] is the global optimization variable. *Center*_*i*_, *Direction*_*i*_ and *Rotation*_*i*_ represent the location of the center of rotation, translation, and rotation angle of the *i*th bone, respectively. As a result, the wing flapping motions can be computed using the proposed optimization algorithm.

The flapping motions of the wing skeleton can be computed using the optimization algorithm. Additionally, in our previous research (Tang et al., 2023), it was found that the scapula and coracoid can be treated as a rigid body to represent the entire *F. peregrinus*. Thus, the frame on the coracoid was treated as the global coordinate, and the flapping motions were represented by the relative rotations of each bone in the global coordinate system. Specifically, the shoulder joint rotation was calculated by the motion of the humerus relative to the scapula. The shoulder and wrist joints were modeled using three types of joints: spherical, universal, and revolute joint. The residuals for each case were then calculated using the optimization algorithm.

### Measurement of flapping motions

The Zhejiang Museum of Natural History provided a specimen of a *F. peregrinus* for this study. The *F. peregrinus* was 0.45 m in length, with a wingspan of 1.2 m and a weight of 0.96 kg. The body and wing structure of the *F. peregrinus* were complete, with no signs of trauma, and all joint movements were flexible. The flapping motions were artificially reconstructed under the professional guidance of ornithologists. Six angle sensors were attached to the wing surface of *F. peregrinus* specimens to document the details of their wing kinematics. Ornithologists approximated the flapping process by manually actuating the wing motions. A total of eight cycles of flapping motion were simulated. To ensure the continuity of the flapping motion, only the second to seventh cycles were selected for study. During the tests, three sensors were mounted on each *F. peregrinus* wing to capture the shoulder joint, elbow joint, and wrist joint rotations (Fig. 3A).

### Originality statement

All data and figures in this study are original, except for those with special references.

### Institutional review board statement

Ethical review and approval were waived for this study due to the *F. peregrinus* in this research was the specimen, provided by Zhejiang Museum of Natural History.

## Supplementary Material

10.1242/biolopen.061859_sup1 Supplementary information
