# Supplementary material for: Morphlight theory inspired by raptors: musculoskeletal modeling and muscle control in Falco peregrinus wing flapping
Source: Biol Open. 2025 Apr 10;14(4):bio061859. doi: 10.1242/bio.061859 (PMC12010916; doi:10.1242/bio.061859)
Supplement: Supplementary information [file biolopen-14-061859-s1.pdf]

Table S1. The mass of the wing bone.

**Bone**	**Mass (g)**
coracoid	0.910
scapula	0.660
humerus	4.034
ulna	2.372
radius	0.700
metacarpus	1.475

Table S2. Residuals for 9 cases.

**number**	**shoulder joint**	**wrist joint**	**residual**
1	S	S	0.5868
2	S	U	0.5872
3	S	R	0.6207
4	U	S	0.5877
5	U	U	0.5881
6	U	R	0.6213
7	R	S	0.5892
8	R	U	0.5932
9	R	R	0.6383
